# Demographics, Histopathology, and Treatment Outcomes of Squamous Cell Carcinoma of the Prostate

**DOI:** 10.1002/cnr2.2156

**Published:** 2024-09-22

**Authors:** Julian A. Gordon, Michael C. Larkins, Vaishnavi Siripurapu, Arjun Bhatt, Melisa Pasli, Kristen Armel, Carol Velez‐Martinez, Anastasios Mitsakos, Aidan Burke, M. Sean Peach

**Affiliations:** ^1^ Brody School of Medicine East Carolina University (ECU) Greenville North Carolina USA; ^2^ Division of Hematology/Oncology, Department of Internal Medicine Brody School of Medicine, East Carolina University (ECU) Greenville North Carolina USA; ^3^ Department of Surgery Brody School of Medicine, East Carolina University (ECU) Greenville North Carolina USA; ^4^ Department of Radiation Oncology Brody School of Medicine, East Carolina University (ECU) Greenville North Carolina USA

**Keywords:** cancer demographics, histology, prostate cancer, SEER database, squamous cell carcinoma

## Abstract

**Background:**

Squamous cell carcinoma of the prostate (SCCP) is a neoplasm that comprises fewer than 1% of all primary prostate cancer diagnoses. Given its rarity, there is a paucity of data regarding the treatment of this disease. The limited literature points to the potential of local therapy in conjunction with chemotherapy to improve patient mortality.

**Methods:**

Using the National Cancer Initiative's Surveillance, Epidemiology, and End Results (SEER) database, a retrospective review of patients diagnosed with primary SCCP between 2000 and 2018 was performed. Patient demographics, tumor characteristics, and patient outcomes based on treatment modality were analyzed. Univariate and survival analyses were conducted with *p* < 0.05 indicating statistical significance.

**Results:**

A total of 66 patients were identified. Five‐year overall survival (5y OS) was 24%; mean and median survival were 2.2 years (1.8, 2.7) and 1.2 years (0.3, 2.1), respectively. Patients with Grade I or Grade II disease had an increased 5y OS of 55% (27%, 83%). In comparison, 5y OS was 13% (−2%, 29%) for patients with Grade III and Grade IV disease (*p* = 0.017). Analysis of 5y OS based on disease histology revealed patients with papillary SCC had a 5y OS of 50% [9.2%, 91%], compared to 21% [9%, 34%] for patients with SCC, not otherwise specified and 0% for those with lymphoepithelial carcinoma (*p* = 0.048). Analysis of 5y OS stratified by treatment modality revealed no statistically significant change with any treatment (surgery, radiotherapy, and chemotherapy). No difference in 5y OS was seen between those treated with radical prostatectomy versus external beam radiation therapy.

**Conclusions:**

The literature on SCCP remains sparse; the rarity of this disease limits analysis. While the investigation undertaken in this paper does not find any change in 5y OS regardless of treatment modality, the variation in 5y OS based on histologic classification of SCCP points to a potential route for the future treatment of this disease.

## 1 Introduction

With the exception of non‐melanoma skin cancer, prostate cancer is the most common male malignancy in the United States as of 2020, with about 201 000 new cases and 33 000 deaths attributed to primary prostate cancer [1]. Diagnosis for primary prostate cancer is most prevalent in men aged 65 to 74 years of age and occurs more frequently in Black Americans compared with other races [2, 3, 4]. Evaluation of disease is most often prompted by elevated prostate‐specific antigen (PSA) and confirmed through prostate biopsy, with other evaluations such as digital rectal examination, bioassays, and imaging used for risk group stratification [4]. The vast majority of primary prostate cancers (up to 95%) are histologically adenocarcinomas [5, 6]. From 2016 to 2020, 70% of all cases of primary prostate cancer diagnoses were at a localized stage, and such disease is often managed with single modality therapies such as surgery or radiotherapy, while locally advanced disease is best addressed with a multimodal strategy through a combined modality of radiotherapy, hormone therapy and/chemotherapy [7]. Guidelines for the treatment of prostate cancer advocate for surveillance or observation in patients with low‐risk disease or limited expected survival, while those with greater expected survival and/or higher‐risk disease may benefit from more aggressive treatment [8, 9].

In contrast to adenocarcinoma, squamous cell carcinoma of the prostate (SCCP) makes up fewer than 1% of all primary prostate cancer cases [10]. Its origin is controversial, but it is thought to originate from squamous cells in the prostatic urethral sphincter or as the result of squamous metaplasia of prostatic acini [11]. Literature consists primarily of case reports: generally, these reports discuss cases in which patients either present with obstructive symptoms similar to other forms of primary prostate cancer [6, 12, 13, 14, 15], or they discuss patients that present with advanced disease, often metastatic and described as aggressive [16, 17, 18, 19]. While no true consensus exists regarding treatment of SCCP, most case reports regard surgical excision in line with the extent of the disease as the preferred treatment modality. Three case reports have described the use of chemotherapy and/or radiation to control SCCP, with Munoz et al. reporting the longest survival time of 5 years after treatment with cisplatin, 5‐flurouracil, and whole pelvis with prostatic bed and adjunctive prostate gland radiotherapy [6, 15, 20]. Given the limited information available in the literature, the benefit of chemotherapy and radiotherapy in the treatment of SCCP is not fully understood.

To date the largest review of SCCP is a review of the US National Cancer Database with 66 patients identified between 2004 and 2015, which compared local and systemic therapy but did not distinguish between radiation and surgery [21]. This analysis was further limited to locoregional disease and did not include sub‐analysis specific to Grade and histology. Patients were divided into treatment groups of various combinations of local therapy, chemotherapy, and radiation, with the ultimate finding that when the disease is localized to the prostate, local therapy in combination with chemotherapy is a reasonable treatment option, which increased median survival by 17 months compared with patients that received only local therapy. Our study seeks to build on previous work by providing an in‐depth sub‐analysis of yet unstudied disease Grade and histological subtype, which may both provide clinically relevant information for diagnosis and management of SCCP as well as its biological origin. We also delineate between radiation and surgical therapy, offering more granular data as afforded by the cancer database SEER. We further investigated the impact of these variables on patient overall survival. Finally, we present a comprehensive listing and classification of case reports related to SCCP.

## 2 Methods

### 2.1 Patient Selection

We conducted this retrospective cohort study from the National Cancer Institute (NCI)'s Surveillance, Epidemiology and End Results (SEER) database [22]. The SEER database is a registry that comprises of deidentified disease course data. It consists of approximately 28% of the US oncology population. We queried the database for all cases of “squamous cell neoplasms” (variable name Histology Recode—broad groupings) of the prostate (variable name Site recode ICD‐O‐3/WHO 2008) between the years 2000 and 2018. Exclusion criteria included patients diagnosed with intraductal carcinomas of the prostate, epithelial histologies not otherwise specified (including cribriform morphologies), and non‐primary tumors.

The variables reviewed were: patient demographics, including age and race, and tumor characteristics, which consisted of Grade, tumor type, and histopathologic characteristics. We analyzed Kaplan–Meier survival outcome curves in the setting of treatment modalities, such as surgical resection, radiation therapy, and systemic therapy for various combinations and orders of the previously mentioned treatment types. Disease stage was obtained by recoding two variables: “CS Extension (2004–2015)” and “EOD 10—extent (1988–2003)”, and was stratified in local, regional, and distant disease.

### 2.2 Statistical Analysis

Analysis of continuous variables was conducted using Student's *t*‐test while bivariate analysis was conducted using hazard ratios (HR). Categorical variables were analyzed using a two‐sided Fisher's Exact Test. Survival analysis was performed via Kaplan–Meier curve generation and log‐rank comparisons. For all statistical analyses a *p* value <0.05 indicating statistical significance and 95% confidence intervals are reported in brackets [CI]. Data were analyzed in SPSS (Version 29.0; Armonk, NY: IBM Corp.).

## 3 Results

### 3.1 Patient Demographics/Overall Survival (OS)

**FIGURE 1:**
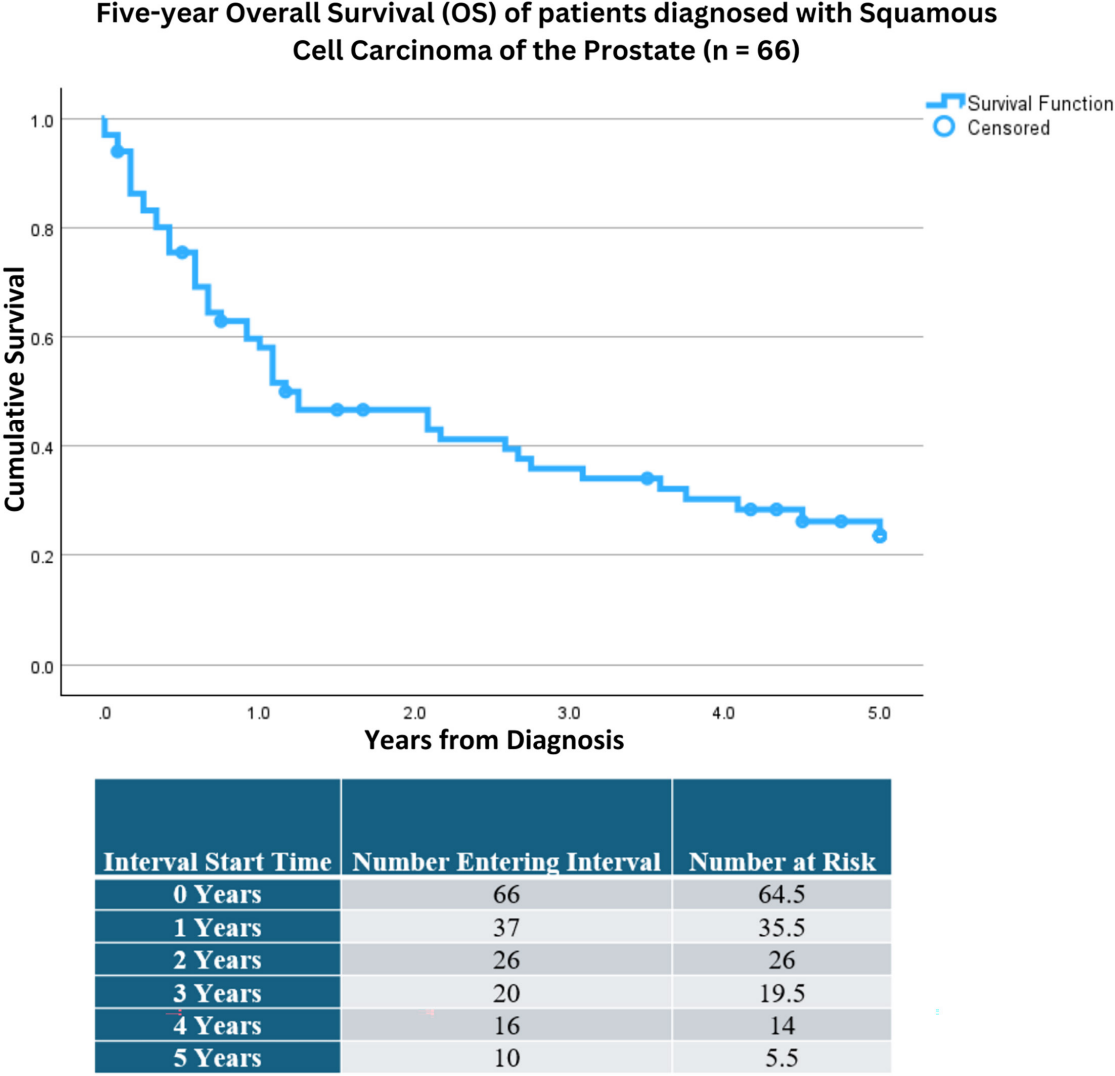
Kaplan–Meier survival curve depicting five‐year overall survival (5y OS) for patients diagnosed with primary squamous cell carcinoma of the prostate (*n* = 66). Patients were identified from the Surveillance, Epidemiology, and End Results (SEER) Program and were diagnosed between 2000 and 2018. 5y OS was ~24% (12%, 35%). Censored cases are represented by a blue circle at time of death. A corresponding life table can be found directly beneath the survival curve depicting the number at risk per year of life.

Sixty‐six patients with SCCP were identified; all were male. Demographics, disease characteristics, and treatment information can be found in Table 1. Five‐year OS (5y OS) was 24% (see Figure 1), while mean and median survival were 2.2 years (1.8, 2.7) and 1.2 years (0.3, 2.1), respectively. Demographically, there were 53 White, 8 Black, and 5 Asian or Pacific Islander patients. No difference in 5y OS was seen with respect to race (*p* = 0.590). The median age at diagnosis was 67 years old, with the youngest and oldest patients diagnosed at 35 and 85 years of age (listed as 85+), respectively. No difference in survival was seen between patients diagnosed before age 65 versus those with age ≥ 65 years at diagnosis (HR = 1.15; *p* = 0.621). It was noted that 63.8% of all patients diagnosed with SCCP had this disease listed as their cause of death.

**TABLE 1:** Breakdown of variables among patients diagnosed with squamous cell carcinoma of the prostate (SCCP) between 2000 and 2018, identified via the Surveillance, Epidemiology and End Results (SEER) Program.

Variable	Count (% of cohort; *n* = 66)
*Age* (*in 5‐year increments*)	
35–39 years	1 (1.5%)
40–44 years	2 (3.0%)
45–49 years	3 (4.5%)
50–54 years	4 (6.1%)
55–59 years	4 (6.1%)
60–64 years	11 (16.7%)
65–69 years	12 (18.2%)
70–74 years	4 (6.1%)
75–79 years	12 (18.2%)
80–84 years	7 (10.6%)
85+ years	6 (9.1%)
*Age* (*diagnosed* <65 *years* vs. ≥65 *years*)	
<65 years	25 (37.9%)
≥65 years	41 (62.1%)
*Race*	
Asian/Pacific Islander	5 (7.6%)
Black	8 (12.1%)
White	53 (80.3%)
*Clinical grade*	
Grade I	4 (6.1%)
Grade II	10 (15.2%)
Grade III	22 (33.3%)
Grade IV	3 (4.5%)
Unknown/incomplete	27 (40.9%)
*Histology*	
SCC, not otherwise specified (NOS)	52 (78.8%)
Keratinizing SCC	5 (7.6%)
Papillary histology^a^	5 (7.6%)
Lymphoepithelial carcinoma	1 (1.5%)
*Stage* (*summary*)	
Local	16 (24.2%)
Regional	15 (22.7%)
Distant	19 (28.8%)
Unknown/incomplete	16 (24.2%)
*Treatment*	
Curative surgery	20 (30.3%)
Radiotherapy (RT)	2 (3.0%)
Chemotherapy (CTX)	3 (4.5%)
Surgery + RT	3 (4.5%)
(Adjuvant RT)	2 (3.03%)
(Neoadjuvant RT)	1 (1.5%)
Surgery + CTX	7 (10.6%)
(Adjuvant CTX)	3 (4.5%)
(Neoadjuvant CTX)	2 (3.03%)
(Both neoadjuvant and adjuvant CTX)	2 (3.03%)
RT + CTX	4 (6.1%)
Surgery + CTX + RT	4 (6.1%)

^a^ Includes ICD‐O‐3 codes 8050/3 (papillary carcinoma, NOS) and 8052/3 (papillary squamous cell carcinoma).

### 3.2 Tumor Characteristics

#### 3.2.1 Clinical Grade

**FIGURE 2:**
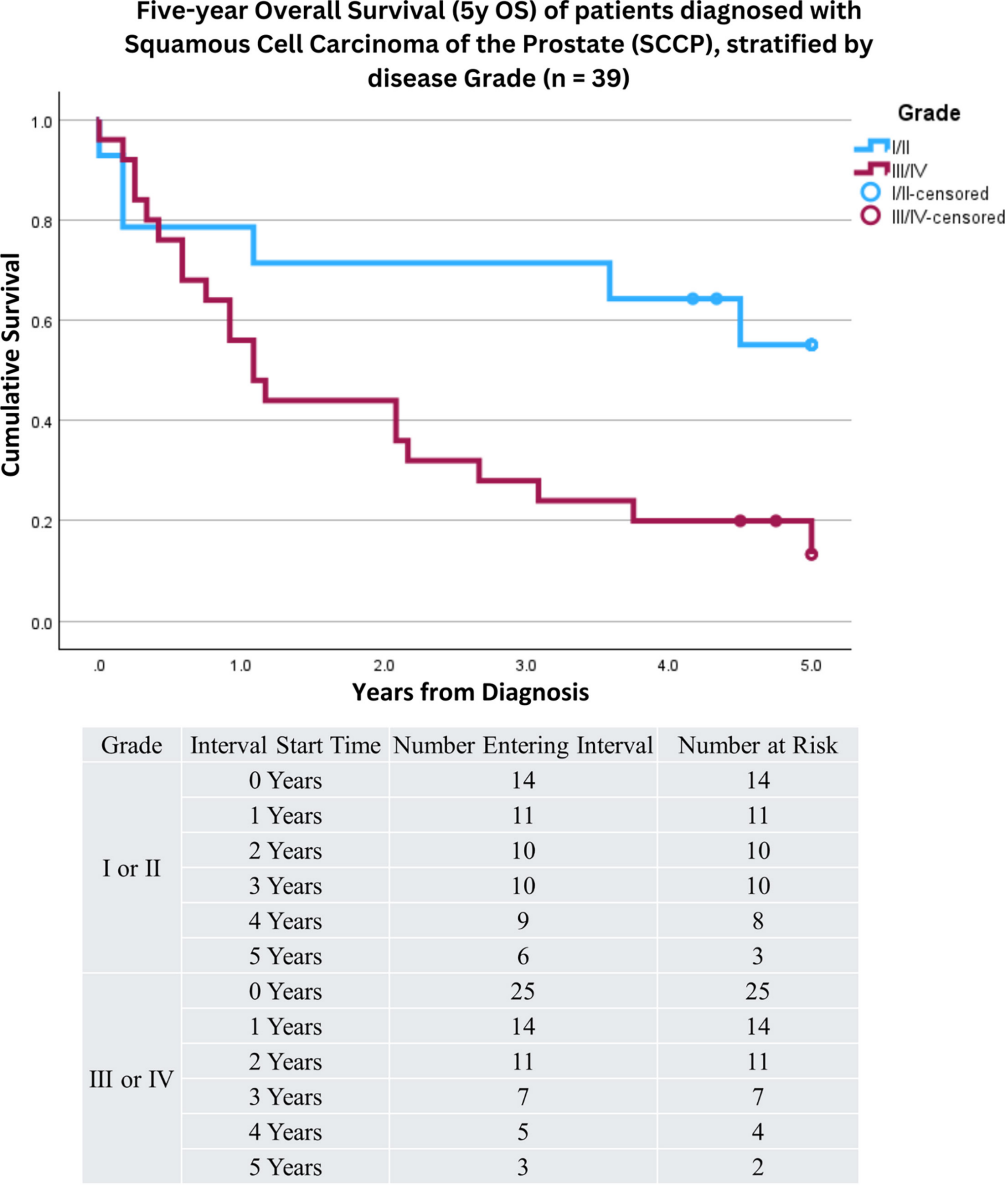
Kaplan–Meier survival curve depicting five‐year overall survival (5y OS) for patients diagnosed with squamous cell carcinoma of the prostate, stratified by disease Grade (*n* = 39). Patients with unknown or incomplete Grading were excluded. Patients with Grade I or Grade II disease saw an increased 5y OS of 55%, compared with patients diagnosed with Grades III or IV disease, who saw a 5y OS of 13% (*p* = 0.017). Patients were identified from the Surveillance, Epidemiology, and End Results (SEER) Program and were diagnosed between 2000 and 2018. Censored cases are represented by a blue circle at time of death. A corresponding life table can be found directly beneath the survival curve depicting the number at risk per year of life.

Cases of SCCP stratified by Grade were as follows: 33.3% were Grade III (poorly differentiated), 15.2%% were Grade II (moderately differentiated), 6.1% were Grade I (well differentiated), and 4.5% were Grade IV (undifferentiated); the remainder had unknown or incomplete entries (see Table 1). Grouping patients with Grade I and Grade II disease and comparing to patients with Grade III and Grade IV disease revealed patients with Grade I or Grade II disease had an increased 5y OS of 55% (27%, 83%). In comparison, 5y OS was 13% (−2%, 29%) for patients with Grade III and Grade IV disease (*p* = 0.017; Figure 2). No difference in disease Grade was seen with respect or patient age (<65 years vs. ≥65 years; *p* = 0.375) or race (*p* = 0.637).

#### 3.2.2 Histologic Characteristics

**FIGURE 3:**
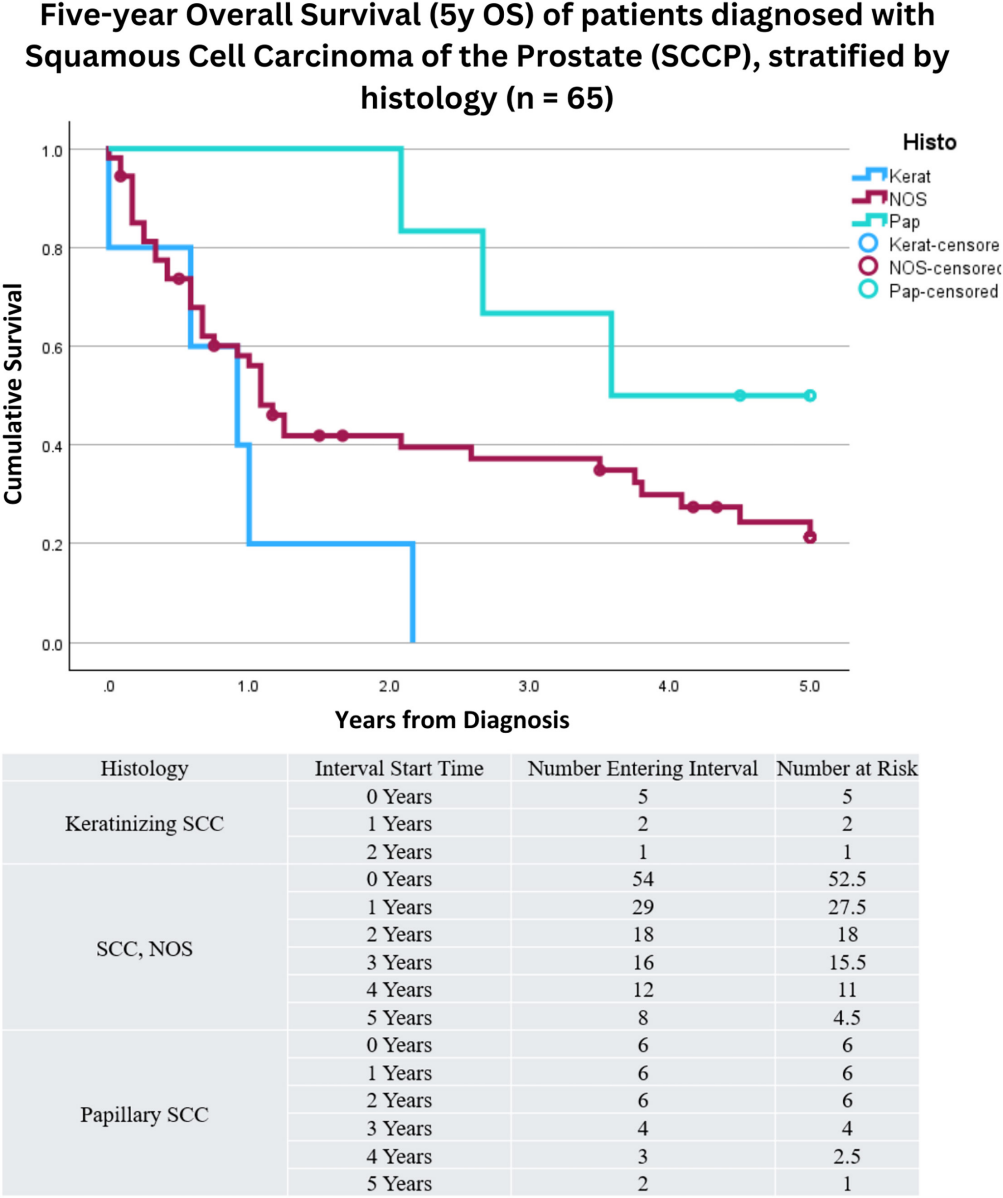
Kaplan–Meier survival curve depicting five‐year overall survival (5y OS) for patients diagnosed with squamous cell carcinoma of the prostate (SCCP), stratified by disease histologic type and excluding one patient diagnosed with lymphoepithelial SCCP (*n* = 65). Patients with papillary SCC had a 5y OS of 50% (9.2%, 91%), compared with 21% (9%, 34%) for patients with SCC, NOS. Furthermore, all patients with keratinizing SCC died within 3 years of diagnosis (5y OS = 0%). Log‐rank comparison among these three histologies demonstrated survival difference (*p* = 0.048). Patients were identified from the Surveillance, Epidemiology, and End Results (SEER) Program and were diagnosed between 2000 and 2018. Censored cases are represented by a blue circle at time of death. A corresponding life table can be found directly beneath the survival curve depicting the number at risk per year of life.

Histologically, 78.8% of cases were considered squamous cell carcinoma, not otherwise specified (NOS). In contrast, 21.2% of cases fell under other histopathological categories such as keratinizing squamous cell carcinoma (7.6%), papillary squamous cell carcinoma (7.6%), and lymphoepithelial carcinoma (1.5%; see Table 1). Excluding the one patient with lymphoepithelial carcinoma, analysis of the 5y OS based on disease histology revealed patients with papillary SCC had a 5y OS of 50% (9.2%, 91%), compared with 21% (9%, 34%) for patients with SCC, NOS (see Figure 3). Furthermore, all patients with keratinizing SCC died within 3 years of diagnosis (5y OS = 0%). Log‐rank comparison among these three histologies demonstrated survival difference (*p* = 0.048). No difference in the distribution of histological subvariants of SCCP were seen with respect to patient age or race (*p* = 1.000 and 0.159, respectively).

### 3.3 Treatment Modalities

A total of 43 patients diagnosed with SCCP went on to receive treatment; a breakdown of our patient population by treatment administered can be seen in Table 1; 23 patients either refused treatment or were not eligible for treatment with curative intent. Overall, 79.1% of patients underwent some type of surgery. 30.3% of these patients received surgery alone, while 21.2% underwent surgery in conjunction with chemotherapy (CTX) and/or radiotherapy (RT). Of the three patients treated with surgery and RT, one was treated with neoadjuvant RT, and two were treated with adjuvant RT. Of the seven patients treated with both surgery and CTX, three were treated with adjuvant CTX, two with neoadjuvant therapy, and two with both neoadjuvant and adjuvant CTX. All patients that did not undergo surgery for treatment of SCCP (due to patient eligibility, refusal, or unknown reason) died within 2 years of diagnosis. Analysis of 5y OS not accounting for disease stage‐stratified by treatment modality revealed no statistically significant change with any treatment: surgical treatment (*p* = 0.82), treatment with RT (*p* = 0.47), and treatment with CTX (*p* = 0.10).

Sub‐analysis in terms of definitive treatment and not accounting for disease stage found that three patients underwent radical prostatectomy (RP) compared with 12 that received external beam radiotherapy (EBRT). Of these 12, 7(58%) received concurrent chemotherapy. No difference was seen in average survival between these two treatment modalities (HR = 1.0; *p* = 1.0). No survival benefit was seen with the addition of chemotherapy to treatment with EBRT (*p* = 0.618). No difference was seen in the rates of RP versus EBRT with respect to race or patient age (*p* = 0.098 and 0.339, respectively).

### 3.4 Disease Stage

**FIGURE 4:**
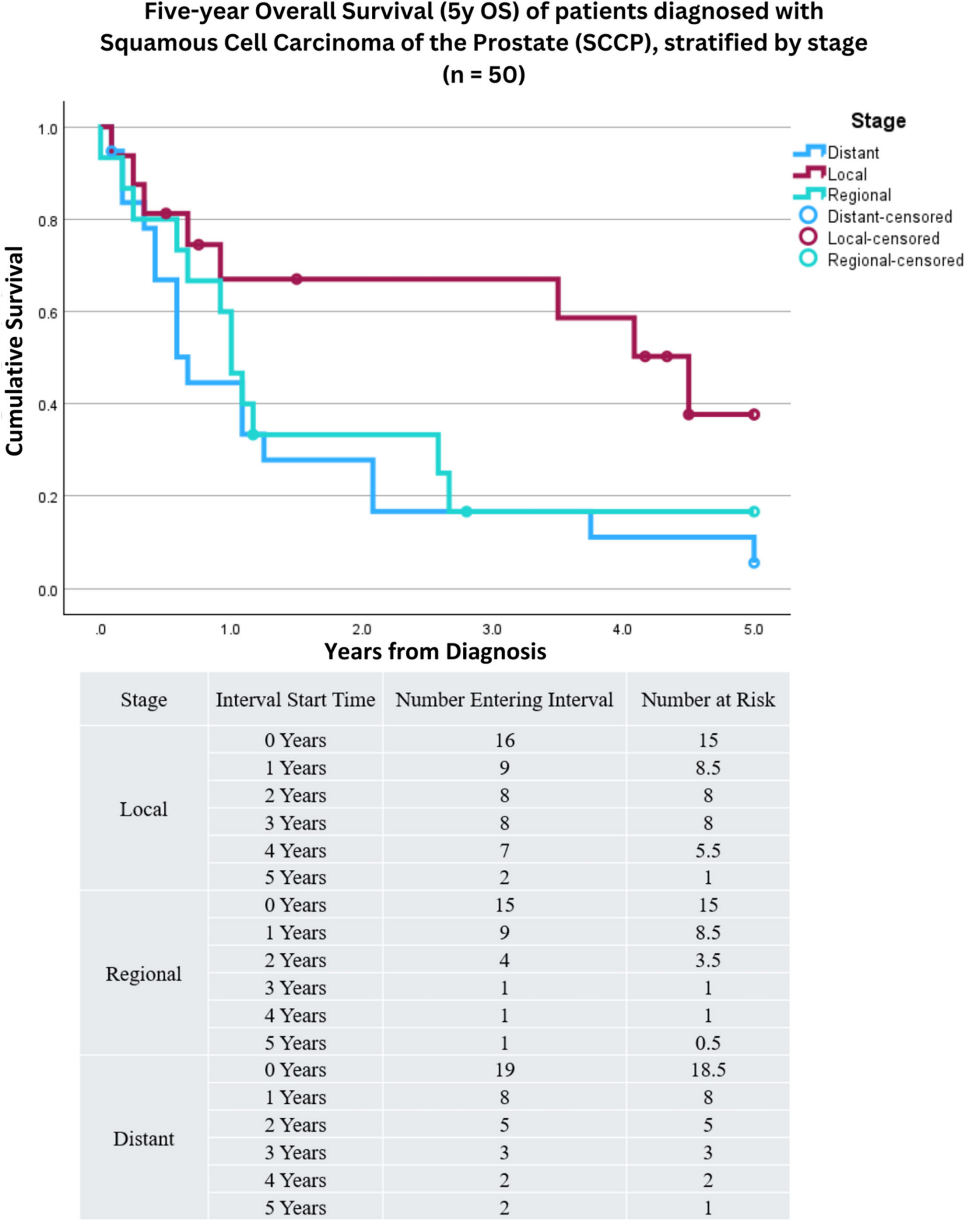
Kaplan–Meier survival curve depicting five‐year overall survival (5y OS) for patients diagnosed with squamous cell carcinoma of the prostate (SCCP), stratified by disease Stage and excluding patients with unknown or incomplete staging (*n* = 50). 16 patients were identified with local disease, 15 with regional, and 19 with distant. Patients with local disease had increased 5y OS (38% [8%, 68%]) compared to those with regional (17% [−4%, 37%]) and distant disease (6% [−5%, 16%]). Log‐rank comparison between summary stages demonstrated those with local disease had increased 5y OS compared to those with either regional or distant disease (*p* = 0.044).

Fifty patients with complete staging information were identified: 16 with local disease, 15 with regional disease, and 19 with distant disease. Five‐year OS stratified by stage can be found in Figure 4; patients with local disease had increased 5y OS (38% [8%, 68%]) compared to those with regional (17% [−4%, 37%]) and distant disease (6% [−5%, 16%]). Log‐rank comparison between summary stages demonstrated those with local disease had increased 5y OS compared to those with either regional or distant disease (*p* = 0.044). Of the 31 patients with local or regional disease, 6 underwent treatment with curative intent (1 [17%] underwent RP and five [83%] received EBRT). Stratification by disease stage and treatment modality did not reveal a difference in 5y OS for surgical treatment, radiotherapy, or chemotherapy when compared to those that did not receive each respective treatment (see Table 2).

**TABLE 2:** Mean survival of stage‐stratified treatment modalities.

Variable	Stage	Mean survival over 5 years (years)	*p*
Chemotherapy (vs. none)	Local	0.9 years [0.9, 0.9] (*n* = 1) versus 3.4 years [2.4, 4.5] (*n* = 15)	*p* = 0.30
	Regional	1.8 years [0.7, 2.8] (*n* = 4) versus 1.6 years [0.6, 2.6] (*n* = 11)	*p* = 0.69
	Distant	1.9 years [0.8, 3.0] (*n* = 11) versus 0.6 years [0.3, 0.9] (*n* = 8)	*p* = 0.08
Radiotherapy (vs. none)	Local	2.6 years [0.0, 5.2] (*n* = 3) versus 3.2 years [2.1, 4.3] (*n* = 13)	*p* = 0.72
	Regional	0.8 years [0.4, 1.1] (*n* = 4) versus 1.8 years [0.8, 2.8] (*n* = 11)	*p* = 0.60
	Distant	2.1 years [0.8, 3.5] (*n* = 6) versus 1.0 years [0.3, 1.8] (*n* = 13)	*p* = 0.16
Surgery (vs. none)	Local	2.8 years [1.4, 4.1] (*n* = 8) versus 3.8 years [0.3, 3.1] (*n* = 8)	*p* = 0.15
	Regional	2.0 years [1.0, 3.1] (*n* = 10) versus 0.6 years [0.2, 1.0] (*n* = 5)	*p* = 0.20
	Distant	1.2 years [0.4, 2.0] (*n* = 11) versus 1.7 years [0.3, 3.1] (*n* = 8)	*p* = 0.95

*Note:* Table depicting the treatment of patients diagnosed with squamous cell carcinoma of the prostate (SCCP) diagnosed between 2000 and 2018 stratified by treatment modality and disease Stage (*n* = 50). Mean survival over 5 years is presented in comparison to the absence of such treatment, with 95% confidence interval and the number in each group provided.

## 4 Discussion

Although case reports and literature reviews on the SCC of the prostate have been previously published, few articles in the literature offer comprehensive insight into the longitudinal course of the disease. The SEER database encompasses a variety of treatment courses and outcomes across multiple states, time periods, and registries, enabling us to fill gaps in the literature with unique, longitudinal views into the courses of rare tumors such as prostate SCC. With this disease being aggressive in nature, particularly when compared with prostate adenocarcinoma, study of this disease have been and continues to be limited.

Given the rarity of this disease, case reports in the literature are sparse. Mohan et al. published a similar table of case reports in 2003, consisting of 65 cases [12]. During our literature review, an updated list of case reports since that date were compiled; these results may be found in Table 3. Notably, no case report in the past 20 years has reported on the histopathologic subtype of SCCP; this histopathologic classification of SCCP may also yield further insight into the origin of the disease. Presently, SCCP is controversially thought to arise from the urethral epithelium of the prostate; alternative proposed sites of origin include the transitional epithelium of periurethral ducts as well as the basal cells of the prostatic acini [13, 14]. Tracing the observed histopathological variants described in this study down to a single originating cell type may be made possible through the continued examination of SCCP; our work suggests this study may have important prognostic implications. Specifically, our analysis demonstrates that patients with papillary SCCP experience a significantly increased 5y OS compared with all other types of SCCP (*p* = 0.048); a quantitative survival difference was especially pronounced when comparing papillary SCCP to keratinizing subtypes. Increased survival of papillary subtypes of SCC have been reported among other cancers (e.g., head and neck SCC) but such an observation has not been noted in the literature regarding SCCP [23]. Continued study may also yield mechanistic information, based on the relative growth rates of keratinizing and papillary cells, that may explain trends described in the current analysis.

**TABLE 3:** Case reports of squamous cell carcinoma of the prostate.

Author	Year published	Treatment	Grade	Stage	Survival timeframe
Munoz et al.	2007	Chemotherapy, radiotherapy	Not specified	III	>5 years
Mohan et al.	2003	Not specified	Not specified	Not specified	Not specified
He et al.	2021	Radiotherapy, surgery	III	Not specified	>9 months
Wang et al.	2012	Surgery, chemotherapy	III	Not specified	>6 months
Onoda et al.	2016	Chemotherapy, radiotherapy	II	III	>2 years
Kaneko et al.	2022	Chemotherapy, surgery	Not specified	IV	Not specified
Hanna et al.	2020	Surgery, chemotherapy	III	IV	Not specified
Malik et al.	2011	Palliative radiotherapy	Not specified	IV	3 months

*Note:* Table showing case reports of patients with primary squamous cell carcinoma of the prostate (SCCP) since the most recent case report review, by Mohan et al. [12]. No case report mentioned the histopathologic characteristics of the SCCP each patient was diagnosed with. Author, year of publication, treatment, disease Grade and Stage, and reported survival timeframe are reported. Staging was reported based on the American Joint Committee on Cancer (AJCC) and Union for International Cancer Control (UICC) guidelines.

An important consideration in the treatment of prostate cancer is the role of RT versus surgery. Current National Comprehensive Cancer Network (NCCN) guidelines for patients with prostate cancer recommend RT (either EBRT or brachytherapy) or RP in the context of expected patient survival and disease risk [9]. Given the aggressive nature and rarity of SCCP, the applicability of such guidelines is uncertain. Our analysis provides some idea of this applicability, namely that while the analysis was limited by a small study population, no difference in 5y OS was seen between RP or EBRT. It was not possible to account for stage in this analysis though, limiting the context in which these results can be interpreted.

Besides the expected bias toward males, the present analysis revealed typical demographic trends for sporadic tumors with respect to age at diagnosis and race. No demographic variable, including age, produced a statistically significant change in survival outcomes. This is consistent with the aggressive nature of the tumor, and the finding from cause of death data that the majority of patients (63.8%) die from causes directly related to this cancer rather than other causes.

### 4.1 Limitations

While the SEER database holds a plethora of data, it does have limitations. Demographics, such as patient age (currently reported as a range) and socioeconomic status, entailing income and education, are missing. The SEER database does not provide information on environmental factors such as tobacco and alcohol use, which have been linked to SCCP and squamous cell carcinoma in general. There is limited information on the pattern of disease spread, including vascular versus perineal infiltration, available in the SEER database, which may be helpful to record as the number of cases accumulates and finer analyses become feasible. In regard to surgery, the SEER database does not provide any information about the procedure specifics that patients underwent or any complications that may have been associated with the procedure. Furthermore, information on palliative treatments is not available via analysis of the SEER database. The same is true for chemotherapy and radiotherapy. Additionally, sub‐analysis of outcomes based on multimodal therapy and outcome stratification by disease stage or extent of surgery was limited by the small number of SCCP cases. Future work may involve addressing these limitations.

## 5 Conclusions

In summary, SCCP is a rare cancer with limited literature available on its demographics, histology, and treatment. Our analysis found significant variation in 5y OS between the various histopathologic subtypes of this disease listed in the SEER database, with patients with papillary SCC having the highest 5y OS of all recorded types. No unimodal treatment of SCCP showed an improvement in 5y OS. Future work should be conducted in order to further investigate the potential effect of histology on disease treatment and mortality, as well as the impact of multimodal therapy.

## Author Contributions

**Julian A. Gordon:** investigation, formal analysis, project administration, writing – original draft, writing – review and editing. **Michael C. Larkins:** formal analysis, project administration, writing – original draft, writing – review and editing, investigation. **Vaishnavi Siripurapu:** writing – original draft, writing – review and editing, project administration, formal analysis, investigation. **Arjun Bhatt:** conceptualization, software, investigation, formal analysis, funding acquisition, project administration, writing – original draft, writing – review and editing. **Melisa Pasli:** investigation, formal analysis, project administration, writing – original draft, writing – review and editing. **Kristen Armel:** investigation, writing – original draft, writing – review and editing, resources, project administration. **Carol Velez‐Martinez:** project administration, validation, supervision. **Anastasios Mitsakos:** project administration, validation, supervision. **Aidan Burke:** project administration, supervision, validation. **M. Sean Peach:** project administration, supervision, validation.

## Ethics Statement

No Institutional Review Board approval was necessary for this project given the data are de‐identified and publicly available.

## Conflicts of Interest

The authors declare no conflicts of interest.

## Data Availability

The data that support the findings of this study are publicly available from https://seer.cancer.gov/.
